# Synergistic efficacy of salicylic acid with a penetration enhancer on human skin monitored by OCT and diffuse reflectance spectroscopy

**DOI:** 10.1038/srep34954

**Published:** 2016-10-10

**Authors:** Qingliang Zhao, Cuixia Dai, Shanhui Fan, Jing Lv, Liming Nie

**Affiliations:** 1 State Key Laboratory of Molecular Vaccinology and Molecular Diagnostics & Center for Molecular Imaging and Translational Medicine, School of Public Health, Xiamen University, Xiamen 361102, China; 2 College of Physics, Shanghai Institute of Technology, Shanghai 201418, China; 3 College of Life Information Science and Instrument Engineering, Hangzhou Dianzi University, Hangzhou, Zhejiang 310018, China

## Abstract

Salicylic acid (SA) has been frequently used as a facial chemical peeling agent (FCPA) in various cosmetics for facial rejuvenation and dermatological treatments in the clinic. However, there is a tradeoff between therapeutic effectiveness and possible adverse effects caused by this agent for cosmetologists. To optimize the cosmetic efficacy with minimal concentration, we proposed a chemical permeation enhancer (CPE) azone to synergistically work with SA on human skin *in vivo*. The optical properties of human skin after being treated with SA alone and SA combined with azone (SA@azone) were successively investigated by diffuse reflectance spectroscopy (DRS) and optical coherence tomography (OCT). Our results revealed that as the SA concentration increased, the light reflectance decreased and the absorption increased. We also found that SA@azone exhibited a synergistic effect on enhancing light penetration and OCT imaging depth. We demonstrated that the combination of DRS and OCT techniques could be used as a noninvasive, rapid and accurate measurement method to monitor the subtle changes of skin tissue after treatment with FCPA and CPE. The approach will greatly benefit the development of clinical cosmetic surgery, dermatosis diagnosis and therapeutic effect inspection in related biomedical studies.

Skin, as an important part of the human body, can be divided into epidermis, dermis and subcutaneous tissue layers. Its primary role is to act as a strong shield to guard the body against extremes of temperature, sunlight, and chemical hazards in the environment. The stratum corneum (SC), as the outmost layer of skin, is a formidable barrier to prevent transepidermal water loss as well as percutaneous absorption of applied drugs^1^,^2^,^3^. Failure in protection of the skin will cause wrinkles, pore bulky, acne spots, yellow calluses or even more serious diseased state such as seborrheic dermatitis and psoriasis^4^,^5^.

Chemical peeling (CP) has been widely explored for facial rejuvenation over the past decade^6^,^7^,^8^,^9^,^10^. Usually, CP can be classified into three categories: superficial, medium-depth, and deep CP. Among which, superficial CP is on predominant demand in clinic^5^. Salicylic acid (SA), one of the superficial facial CP agents (FCPA), has exhibited excellent desmolytic and comedolytic properties^9^,^10^,^11^,^12^,^13^, and thus, are widely employed to treat skin diseases such as acne vulgaris, photodamage, freckles and lentigines^6^. The efficacy of SA peeling in Fitzpatrick skin types I-III, V and VI has been well documented^12^. Marczyk *et al*. found that SA had greater sebumetric effect than pyruvic acid^9^.

Although the desquamation effect of SA has been widely used as a superficial FCPA on the stratum corneum and the epidermal layer, the dose used is usually high between 20–30%^13^. The risk of salicylism is found to be low in intact skin during chemical peeling for dark-skin people, however, other adverse effects are still under debate in clinic^14^,^15^,^16^. The concentration of actual SA in the cosmeceutical products was below 3% in North America and Europe^17^. In China, Ministry of Health regulates strictly for the usage of SA in cosmetics with an allowable concentration lower than 2%^18^. However, the SA content at this level usually could not satisfy the purpose of facial rejuvenation and usually require repeated dose^16^. Therefore, to optimize the efficacy of SA with minimal dose is of vital significance in clinical facial applications.

Recently, various skin penetration enhancement strategies, including physical and chemical methods, have been conducted to accelerate drug delivery across the SC. In this study, we use azone as chemical permeation enhancer for effective SC pealing agent delivery into skin. FCPAs can effectively cause the morphological structure and optical property variations of skin tissue. However, the exploration of enhanced skin delivery ability by azone is rare in human model. Therefore, a reliable and non-invasive measuring method is highly desirable to real-time monitor those properties changes. It not only enables dermatologists to comprehensively understand the effect of FCPA treatment on patient skin, but also allows for long-term monitoring. By virtue of these, dermatologist can timely and accurately adjust treatment strategy such as drug dose, concentration, and treatment duration.

Optical spectrometry has been employed as a non-invasive, simple, and fast method for biomedical study of dermatoses^19^. This technology is mainly based on the differences of various endogenous substances with characteristic optical bands^20^. Optical coherence tomography (OCT) is a high-resolution, real-time imaging technology that relies on the interference signal from the back-reflected light of sample and reflected light of reference arms^21^. As a result, combining high-sensitivity detection of spectrometry with obvious morphologic visualization of OCT can potentially provide comprehensive information of biological tissues. The cooperation of these two techniques will allow for disease diagnosis as well as real-time treatment monitoring, and ultimately, improve disease management with reduced risks for the patients.

In this work, it is the first time to systematically evaluate the dose effect of SA on optical properties on human skin *in vivo*. We also explored the synergistic effect of SA combined with azone (SA@azone) with diffuse reflectance spectroscopy (DRS) and OCT. The dynamic optical spectrum changes and OCT images of skin were acquired in real time. The variations of diffuse reflectance intensity (RI). OCT signals and 1/e light penetration depth were quantitatively analyzed with the aim to optimize the treatment effect. Our study indicated that SA permeability can be significantly enhanced by azone at low concentration. Morphologic imaging and optical properties of skin tissue also demonstrated that the SA@azone could maximize the chemical facial rejuvenation with minimum risks.

## Results and Discussion

**Figure 1:**
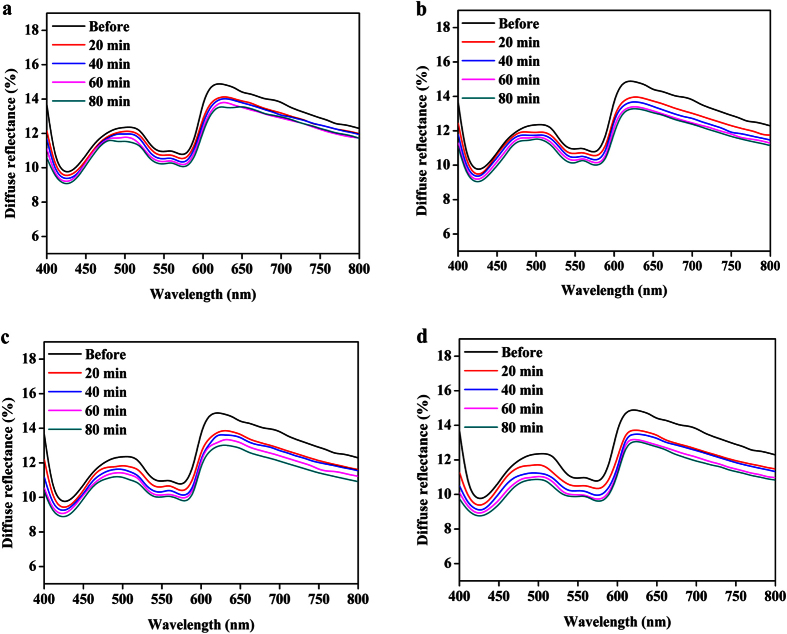
Dynamic spectral changes of the human skin treated with SA. (**a**–**d**) The spectral changes of skin before and after application of the S_1_, S_2_, S_3_ and S_4_ at the different time intervals measured from 400 to 800 nm, respectively.

Dynamic alterations of spectra features and images of human skin before and after being applied with SA and SA@azone at different time intervals were investigated by DRS and OCT. Non-invasive and rapid acquisition of the changes of skin optical properties caused by chemical agents are very meaningful for beautician and dermatologist. Figure 1 shows the DR spectra shift of human skin from 400 to 800 nm at the time intervals of 0, 20, 40, 60 and 80 min, before and after application of S_1_, S_2_, S_3_, S_4_ (See Materials Section), respectively. It can be seen that all the DR spectra in S_1_-S_4_ have similar profiles with almost the same change trends, which gradually decreased with time. However, the changes of the spectra decline span were obviously different in Fig. 1. The higher concentration group showed greater DR decline than that of lower concentration at the same time intervals. The greatest decrease in DR occurred in S_4_ group, as showed in Fig. 1d. The DR signal intensity change between the control and four groups (S1-S4) after 80 min at 580 nm was shown in Supplementary Fig. S1.

**Figure 2:**
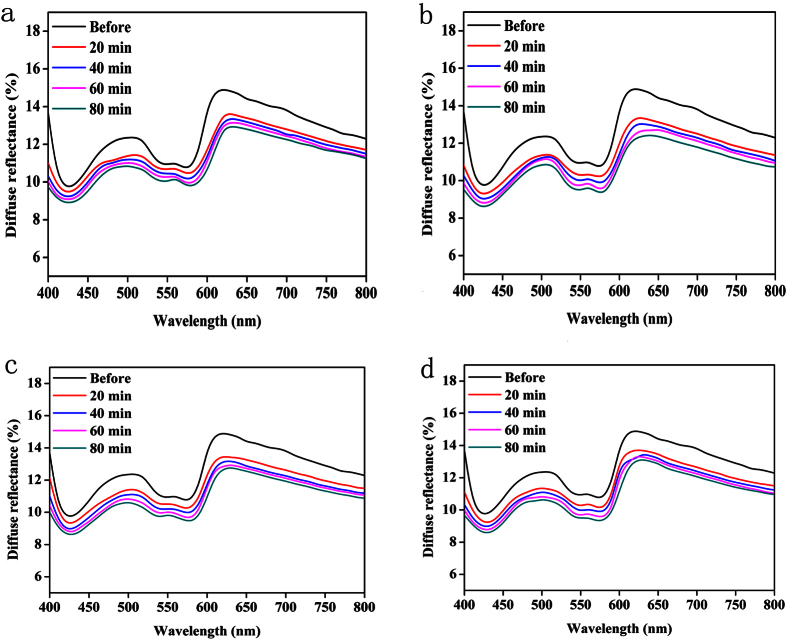
Dynamic spectral changes of the human skin treated with SA@azone. (**a**–**d**) The spectral changes of skin before and after application of the S_1_@A, S_2_@A, S_3_@A and S_4_@A at the different time intervals measured from 400 to 800 nm, respectively.

Figure 2 shows the DR spectra shift of the human skin from 400 to 800 nm before and after application of S_1_@A-S_4_@A (See Materials Section) at the same time interval as shown in Fig. 1. Although their diffuse spectral characteristics did not change much, the DR spectra presented a remarkable reduction (Fig. 2) under azone synergy compared with the SA alone groups (Fig. 1).

**Figure 3:**
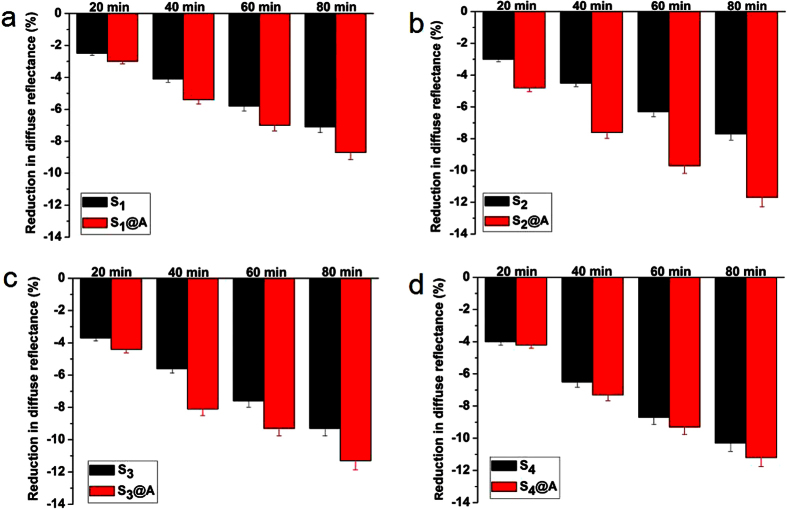
Diffuse reflectance reduction of human skin at 420 nm. Representative diffuse reflectance reduction of human skin treated with (**a**) S_1_ and S_1_@A, (**b**) S_2_ and S_2_@A, (**c**) S_3_ and S_3_@A, (**d**) S_4_ and S_4_@A at different time intervals, respectively.

**Figure 4:**
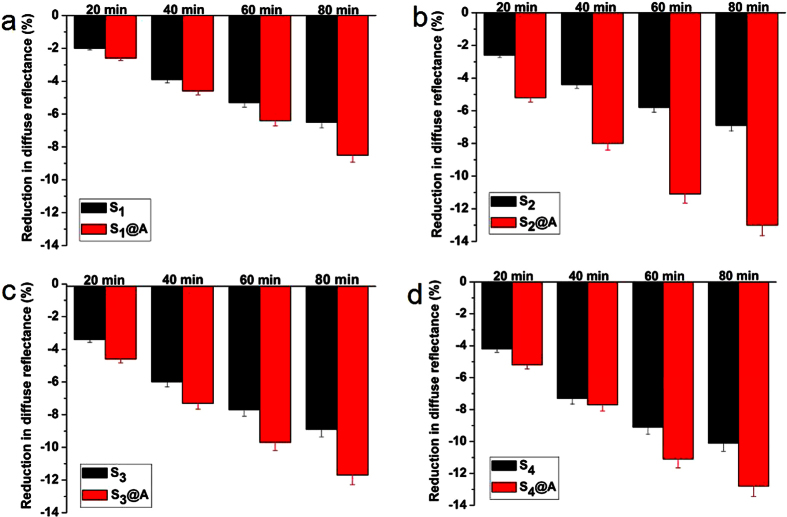
Diffuse reflectance reduction of human skin at 540 nm. Diffuse reflectance reduction at 540 nm for human skin after treatment with (**a)** S_1_ and S_1_@A, (**b**) S_2_ and S_2_@A, (**c**) S_3_ and S_3_@A, (**d**) S_4_ and S_4_@A at 20, 40, 60 and 80 min, respectively.

**Figure 5:**
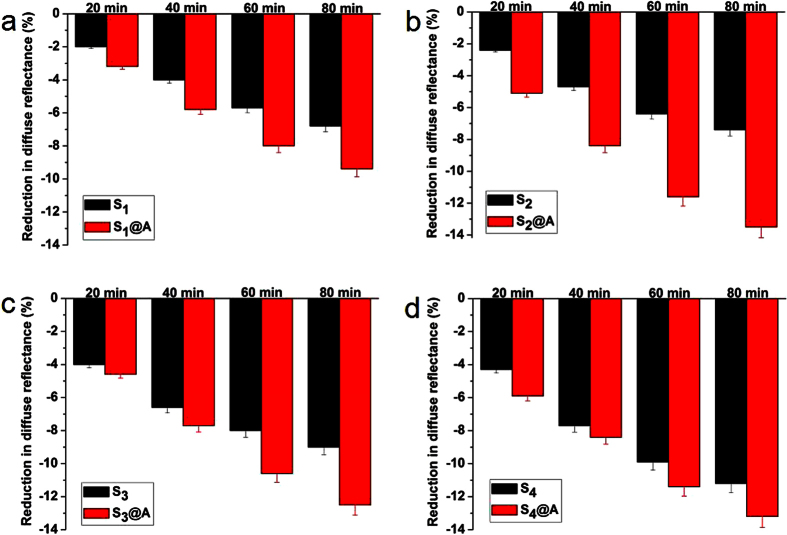
Diffuse reflectance reduction of human skin at 580 nm. Reduction in diffuse reflectance at 580 nm for human skin after treatment with (**a**) S_1_ and S_1_@A, (**b**) S_2_ and S_2_@A, (**c**) S_3_ and S_3_@A, and (**d**) S_4_ and S_4_@A at 20, 40, 60 and 80 min, respectively.

To quantify the optical characteristic variations of skin resulted by S_1_-S_4_ and S_1_@A-S_4_@A, the reduction of DR was calculate at three characteristic wavelengths 420, 540 and 580 nm in the different time points. The DR variations were calculated in Figs 3, 4, 5, respectively. As shown in Figs 3, 4, 5, the DR of S_1_@A-S_4_@A mixed solution reduced more significantly than that of S_1_-S_4_ alone applied on skin tissue at same wavelengths. The DR decreased approximately 2.5%, 4.1%, 5.8% and 7.1%; 3%, 4.5%, 6.3% and 7.7%; 3.5%, 5.6%, 7.6% and 9.3%; 4%, 6.5%, 8.7% and 10.3% at 20, 40, 60 and 80 min after treatment with S_1_, S_2_, S_3_ and S_4_ alone at 420 nm, while in S_1_@A-S_4_@A groups, DR decreased 3%, 5.4%, 7% and 8.7%; 4.8%, 7.6%, 9.7% and 11.7%; 4.4%, 8.1%, 9.3% and 11.3%; 4.2%, 7.3%, 9.3% and 11.2% at the same timeline in Fig. 3.

At 540 nm, the DR decreased approximately 2%, 3.9%, 5.3% and 6.5%; 2.6%, 4.4%, 5.8% and 6.9%; 3.4%, 6%, 7.7% and 8.9%; 4.2%, 7.3%, 9.1% and 10.1% at 20, 40, 60 and 80 min after being treated with S_1_-S_4_ alone, while the DR reduction in S_1_@A-S_4_@A groups was 2.6%, 4.6%, 6.4% and 8.5%; 5.2%, 8%, 11.1% and 13%; 4.6%, 7.3%, 9.7% and 11.7%; 5.2%, 7.7%, 11.1% and 12.8% DR reduction, respectively (Fig. 4). At 580 nm, the DR decreased approximately 2%, 4%, 5.7% and 6.8%; 2.4%, 4.7%, 6.4% and 7.4%; 4%, 6.6%, 8% and 9%; 4.3%, 7.7%, 9.9% and 11.2% at 20, 40, 60 and 80 min after being treated with S_1_-S_4_ alone. Correspondingly, 3.2%, 5.8%, 8% and 9.4%; 5.1%, 8.4%, 11.6% and 13.5%; 4.6%, 7.7%, 10.6% and 12.5%; 5.9%, 8.4%, 11.4% and 13.2% shrinkage were obtained in S_1_@A-S_4_@A groups, respectively (Fig. 5). Those experimental results of DR decreased at 420, 540 and 580 nm showed good agreement with the declined DR spectra in Figs 1 and 2. SA, which is miscible with epidermal lipids and sebaceous gland lipids, possessed keratolytic and desmolytic effect on tissue, leading to the swelling and softening of SC^9^,^10^,^13^,^22^.

We found that the greatest DR decrease occurred in S_4_ group at 20, 40, 60 and 80 min time intervals, which was approximately 1.6-, 1.59-, 1.5- and 1.45-fold; 1.33-, 1.44-, 1.38- and 1.34-fold; 1.08-, 1.16-, 1.14- and 1.1-fold of S_1_, S_2_ and S_3_ at 420 nm, respectively. For 540 nm, S_4_ was about 2.1-, 1.87-, 1.72- and 1.55-fold; 1.62-, 1.66-, 1.57- and 1.46-fold; 1.24-, 1.22-, 1.18- and 1.13-fold of S_1_, S_2_ and S_3_, respectively. For 580 nm, S_4_ was about 2.15-, 1.93-, 1.74- and 1.65-fold; 1.79-, 1.64-, 1.55- and 1.51-fold; 1.08-, 1.17-, 1.24- and 1.24-fold of S_1_, S_2_ and S_3_, respectively. These results may be owing to the increases of lipid disorder in SC and thus increasing the SA diffusivity with azone combination. The results not only revealed that the DR decreased linearly with SA concentration, but also implied that light transmission significantly increased in skin with SA concentration increment, which suggested SA at higher concentration had greater desmolytic ability on the SC^23^,^24^. The DR values decreased at different SA concentration for S_2_ and S_4_ at 540 nm, respectively (Supplementary Fig. S2).

The dose of SA penetration into deeper tissue can be raise by disordering the SC lipids, thus the keratolytic ability of SA also can be improved. Azone has been demonstrated to induce separation and reduce barrier function in the SC lipid domains, and further exert its synergistic effect with FCPA on transdermal drug delivery in skin^25^,^26^. We also observed the coincident synergistic effect on the reduction of DR in Figs 1 and 2. Meanwhile, the synergistic efficacy was remarkably different for S_1_@A-S_4_@A groups. The greatest decrease in DR was not in S_4_@A but in S_2_@A group since the linear concentration dependent effect occurred in SA alone group was not applicable for synergy group. For 420, 540, and 580 nm, the implementation of S_2_@A leads to the 1.6-, 1.69-, 1.54- and 1.52-fold; 2-, 1.82-, 1.91- and 1.88-fold; 2.13-, 1.79-, 1.81- and 1.82-fold decrease on DR after 20, 40, 60 and 80 min than S_2_ alone group, respectively.

In comparison to S_4_, S_2_@A was about 1.2-, 1.17-, 1.11- and 1.14-fold; 1.24-, 1.1-, 1.22- and 1.29-fold; 1.19-, 1.09-, 1.17- and 1.21-fold at 420, 540 and 580 nm, respectively. These numbers showed that the cutaneous permeation ability of S_2_@A was not only higher than S_2_ alone, but also greater than S_4_ group. Azone has been certified as a non-irritant, non-toxic, safety and effectiveness to human skin^27^. Furthermore, azone at 1% concentration was found to achieve the same efficacy as a typical transdermal drug at 10% concentration^28^. Therefore, the greatest DR decrease in S_2_@A may be attributable to the formulation of SA and azone at equal proportion mixture, which indicated that the two components are transported through the same microenvironment of the skin.

**Figure 6:**
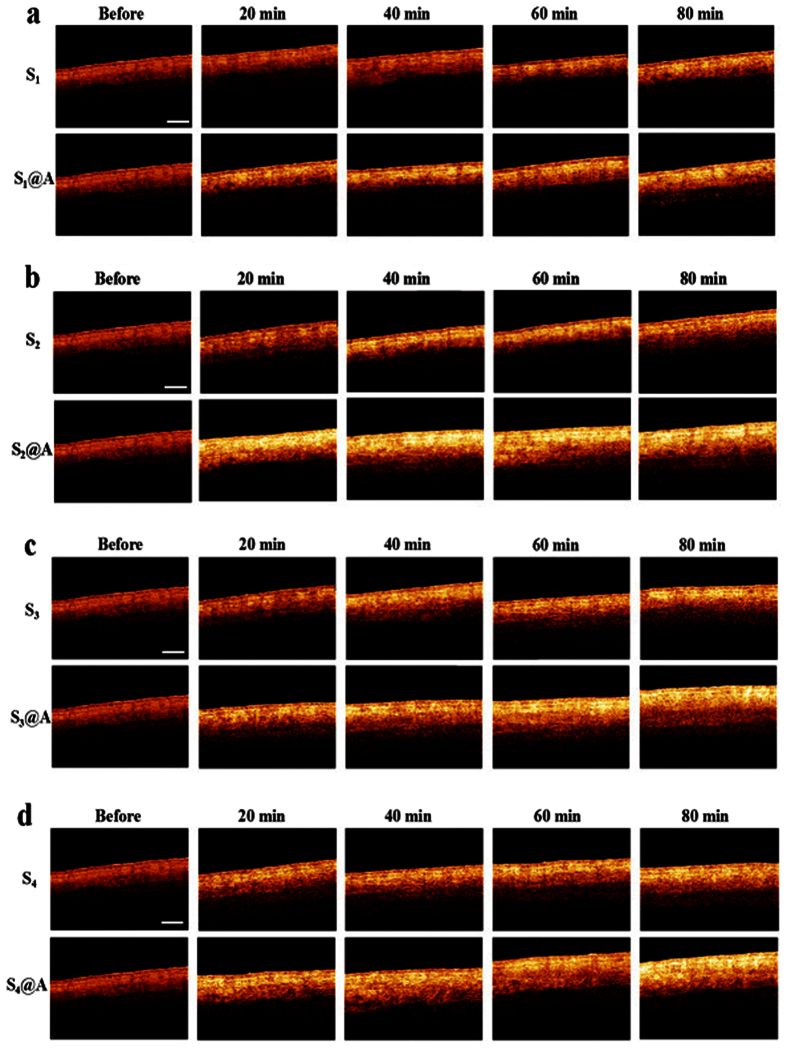
2-D OCT images of human skin tissue. OCT images of skin tissue before and after treatment with (**a**) S_1_ and S_1_@A, (**b**) S_2_ and S_2_@A, (**c**) S_3_ and S_3_@A, (**d**) S_4_ and S_4_@A at 20, 40, 60 and 80 min, respectively. Scale bar: 1 mm.

Dynamic 2-D skin tissue images at different time intervals were obtained with OCT to investigate the penetration enhancement effect (PEE) of 1% azone with SA at different concentrations on the skin. Figure 6 shows the OCT images of skin before and after treated with SA alone and SA@azone at the time intervals of 0, 20, 40, 60 and 80 min, respectively. As shown in Fig. 6, the epidermal structure of skin appears compact and hierarchical before treatment. Structural clarity of tissue and imaging depth of OCT can be improved by S_1_-S_4_ treatment with time. The increase in imaging depth was mainly due to the SA disrupting cellular junctions of SC, the intercellular cohesiveness of corneum cells gradually reduced with the SA permeating. Subsequently, the keratinocytes were rapidly detached from epidermis^16^. Thus, more photons can cross the SC barrier into the inner skin tissue with the increasing intercellular space. More details about SA mechanism can be found in literatures^16^,^29^.

It also can be seen from Fig. 6, there is a remarkable difference in skin treated with SA alone and SA@azone at different concentrations. SA@azone groups revealed stronger optical intensity and deeper imaging depth than the SA alone groups. Figure 6(a–d) show that the structure of skin becomes looser and the imaging depth of OCT has significant enhancement in S_1_@A-S_4_@A at the same time intervals, respectively. This phenomenon caused by the PEE of azone was through interaction with the lipid domains of the SC, which increased skin absorption by reversibly damaging or altering the physicochemical nature of the SC to reduce its diffusional resistance^29^,^30^,^31^. To demonstrate the difference in the texture and structure of the skin after being treated with S_1_-S_4_ and S_1_@A-S_4_@A, hematoxylin and eosin (H&E) histology (10-fold magnification) of fresh abdominal porcine skin was implemented, as described in Supporting Information.

The compactness of skin was destroyed and the liquidity of lipid in the horny layer was boosted with azone synergy, both of which effectively promoted the absorption of SA on skin. At the same time, light transmission can be increased under azone penetration enhancing and convection effects through fluidity of the lipids of skin SC^32^. Certainly, we also can see from the Fig. 6a–d that the improvement of OCT imaging depths is not extremely significant with the azone synergy. However, we can find that the light penetration and imaging depth of OCT is obviously improved after topical application of agents (Fig. 6). The difference in penetration depth between DRS and OCT could be mainly due to different detection light sources. DRS system uses an incoherent light source while OCT employs low-coherence laser. Furthermore, OCT has longer central wavelength than DRS. Long wavelength light has deeper penetration depth than short wavelength.

The optical characteristic changes can indirectly reflect the disrupting cellular junction caused by the agents. In addition, human palm skin is compact and tough, possessing high scattering and low absorption properties, which may be another reason for non-obvious OCT results. However, these factors do not affect the purpose and conclusion of this study. Experiments on nude mice will have considerable results^33^. A direct peeling effect investigation with high-concentration SA and azone on mice skin disease model using DRS and OCT is needed in future.

The solution containing S_2_@A was much more effective than that in S_4_ although both have the same osmolarity. CPEs often work well when used together with FCPA and exhibit synergistic effects in increasing the diffusion of the chemical drug entering the skin. These synergistic mixtures included azone and fatty acids, terpenes and PG, *etc*^33^. Besides, azone can effectively enhance the skin transport of a wide variety of drugs including steroids, antibiotics and antiviral agents reported in previous study^34^.

**Figure 7:**
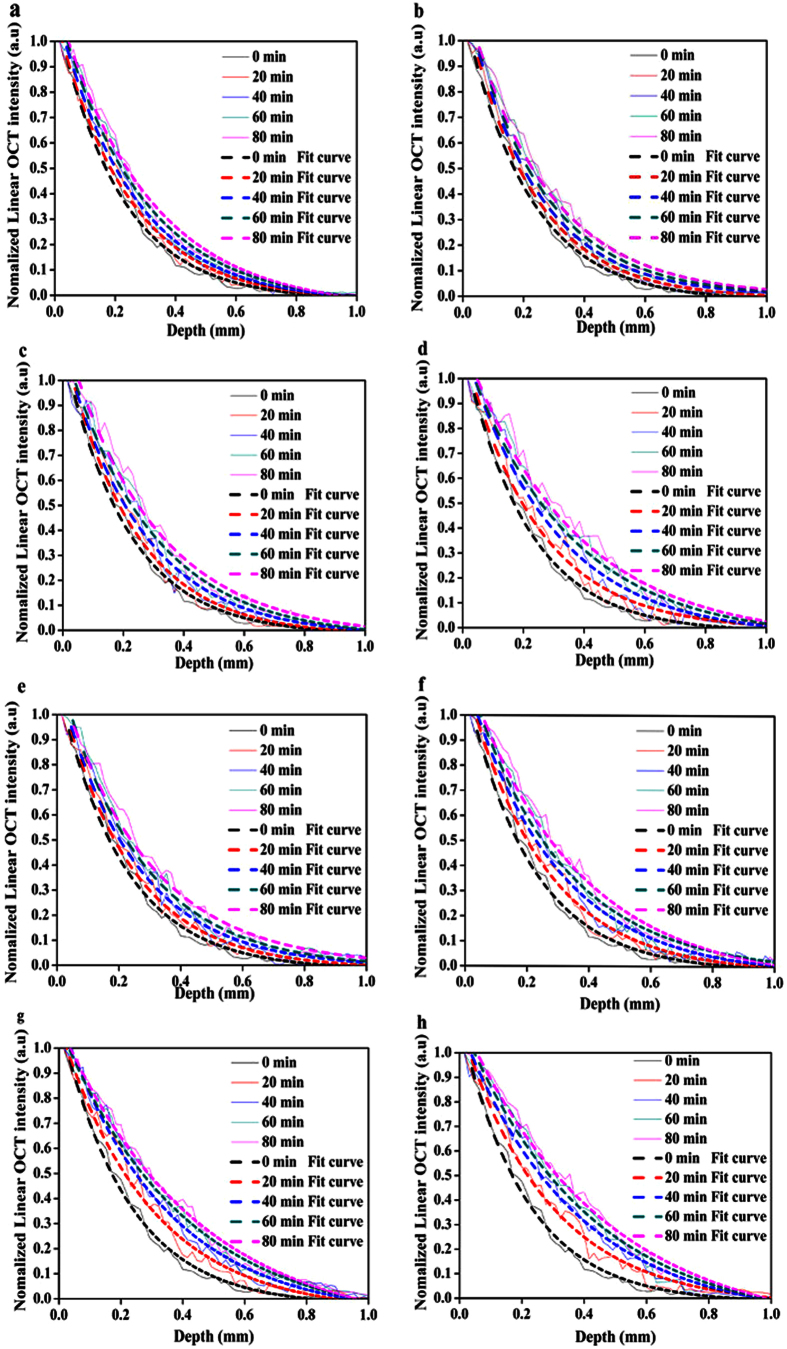
Normalized OCT intensity profiles of human skin. (**a–d**) OCT in-depth reflectance profiles for the human skin topically applied with S_1_-S_4._ (**e–h**) S_1_@A-S_4_@A at 0, 20, 40, 60 and 80 min, respectively.

In our experiment, OCT in-depth reflectance profiles for the human skin topically applied with S_1_-S_4_ alone and S_1_@A-S_4_@A at the time intervals of 0, 20, 40, 60 and 80 min are quantitatively presented in Fig. 7. It can be seen that a similar trend was observed in the S_1_-S_4_ groups. The intensity of the OCT signal gradually increased from the upper layers caused by increase in SA contents over time. This was mainly due to the intercellular cohesiveness of the horny cells reduced with SA diffusion, and then the stratum corneum cells rapidly became detached^16^. Therefore, the light can more easily penetrate from surface into deep tissue of the skin.

The degree of change in the OCT signal intensity was also different in S_1_-S_4_ groups, the high concentration yield a much broader OCT signal enhancement profile in Fig. 7c,d than those observed in Fig. 7a,b at different time intervals. As expected, Fig. 7e–h indicate the intensity of the OCT signal is greatly enhanced after being treated with S_1_@A-S_4_@A. Those results also demonstrated that azone can increase light penetration. The images in Fig. 6 obtained at 20, 40, 60 and 80 min after S_1_@A-S_4_@A treatment shows a significant increase in imaging depth. The S_1_-S_4_ groups without azone show much less enhancement compared with S_1_@A-S_4_@A groups.

**Figure 8:**
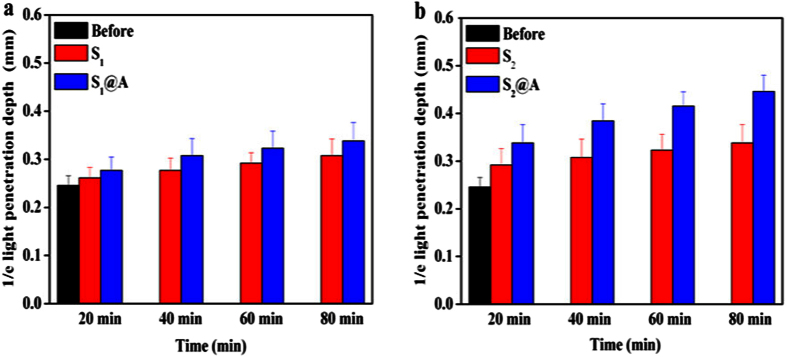
The dynamic changes of 1/e light penetration depth of human skin. (**a**) The changes of light penetration depth of human skin treated before and after S_1_ and S_1_@A, (**b**) S_2_ and S_2_@A at different time intervals, respectively.

In order to quantify the penetration depth enhancement by SA@azone combination, we studied the 1/e light penetration depth changes from graphs of normalized OCT signal intensity at four time intervals. An optimal exponential fit curve covering epidermis and dermis in depth was applied to the averaged and normalized signal intensity data from which the corresponding light penetration depth was derived^34^. As can be seen from Fig. 8a, the corresponding relative 1/e light penetration depth is limited for the skin tissue with the S_1_ alone. The minimum is 0.26 ± 0.02 mm and the maximum is 0.31 ± 0.03 mm (p < 0.05). In contrast, the minimum of S_1_@A is 0.28 ± 0.02 mm and the maximum is 0.34 ± 0.03 mm (p < 0.05), respectively. However, the relative 1/e light penetration depth for S_2_ groups has a minimum of 0.29 ± 0.03 mm and maximum of 0.34 ± 0.04 mm. But for S_2_@A, the minimum is 0.34 ± 0.04 mm and maximum is 0.45 ± 0.03 mm, respectively, as shown in Fig. 8(b). In all cases, the differences were significant statistically (p < 0.05). The increase in 1/e light penetration depth caused by S_1_ and S_2_ solutions is less than those of S_1_@A and S_2_@A solutions in Fig. 8.

The reason could be that azone molecules allow interacting directly with skin lipids and thus generating a more fluid environment^33^, assisting SA to eliminate SC resistance. Once the skin absorption was improved with azone synergy, it will prompt SA penetrates the epidermis into the dermis. Based on the above results, we found that this synergistic method employing azone mixture offered a new pathway to overcome the concentration limitations of individual SA in enhancing transdermal agent transmission. Such findings not only confirmed this efficacious strategy, but also provided an available approach highly expandable to other types of FCPA and CPEs. More importantly, we successfully acquired the change of optical properties induced by SA alone and SA@azone mixed formulation at different concentrations on human skin tissue *in vivo*. Given the effect of slight movement, skin color of subjects in our experiment, further studies are required to investigate the mechanisms of this synergy in the near future. More CPEs and FCPA synergy on dermatosis of nude mice model are our next step work. Meanwhile, the therapeutic effect and systematic toxicity study of FCPA and CPEs synergy at high concentration on mice skin disease model by photodamage are also underway.

It should be noted that our experiments were performed on human palm *in vivo*. Although the palm was fixed gently with adhesive tape, the tiny motion may be existent. In addition, although all the measured on the same skin color Asian people, the gender and age are different, which may also have a little influence on the results. Therefore, an *in vitro* investigation is needed to identify these differences on the measurement.

## Conclusions

In this work, we have studied the change of optical properties induced by SA alone and SA@azone mixed formulation at different combinations on human skin *in vivo* using DRS and OCT. The results show that the desmolytic ability of SA at a relatively low concentration on human skin could be significantly enhanced by azone. Meanwhile, concentration dependent effect of SA on optical properties on skin has also been verified. The experimental results suggested that this new formulation possessed highly synergistic efficacy and was safe and well tolerated on subjects. In addition, DRS and OCT can be applied as powerful tools for fast and non-invasive diagnosis of dermatoses and therapeutic monitoring in clinical dermatology. Our study implied that such formulation may also be considered by commercial cosmetics to improve the exfoliation ability of SA, retardation skin wrinkles, remaining skin vitality.

## Materials and Methods

The schematic of our DRS and OCT detection systems is illustrated in Fig. S3, which consists of a portable spectral detection system and a time-domain OCT system. All the experiments were conducted in accordance with the guidelines of Institutional Review Board at Xiamen University and were approved by Institutional Review Board at Xiamen University. Human experiments were performed on 27 volunteers (14 men and 13 women) aged from 20 to 28 years old with a mean age of 25. All of they were in health situation without any medication. Human experiments were approved by Human Research Ethics Committee of Xiamen University. Informed consent was obtained from all the subjects. Reflection spectra measurement and OCT imaging was conducted on a circle with ~1 cm diameter on the right palm skin. The experimental subjects were divided into two groups: 0.5%, 1%, 1.5% and 2% SA solutions marked as S_1_, S_2_, S_3_ and S_4_, respectively. The others is 0.5%, 1%, 1.5% and 2% SA combined with 1% azone mixture solution (SA@azone) marked as S_1_@A, S_2_@A, S_3_@A, and S_4_@A, respectively. Each concentration group was composed of three independent experiments.

SA was purchased from Sigma Co, Ltd. Azone was purchased from Shanghai Aladdin Industrial Corporation, China. Azone was employed typically at a concentration between 0.1% and 5%^30^. SA was dissolved in distilled water with a thermostatic water bath and the S_1_, S_2_, S_3_ and S_4_ solutions were prepared for later *in vivo* experiments. The mixture solutions of S_1_@A, S_2_@A S_3_@A and S_4_@A used in this study were diluted 10-fold from the mixing solutions of 5%, 10%, 15% and 20% SA and 10% azone with distilled water, respectively.

### Spectroscopic Measurements

Diffuse reflection spectra measurements were carried out on a fiber-optic spectrometer USB 4000 (Ocean Optics, USA) with a Tungsten halogen light source LS-450 (Ocean Optics, USA) over the spectral range from 200 to 1100 nm. The spatial resolution of the system is ~0.4 mm. This spectrometer was equipped with a 3648-element Toshiba linear CCD array that had a spectral resolution of 6 nm in conjunction with the 400 μm-diameter optical fiber light guide. The spectra were calibrated against a diffuse reflection standard of BaSO_4_ with a smooth surface. All the spectrum data were recorded by using the Spectrasuite®Software and stored in a computer. More instrumental details can be found in our previous publication^35^.

Before the experiment, all the subjects were asked to adapt to the darkroom situation and calm their emotion in the laboratory. Then the surface skin of the palm was cleaned using wetting cotton swabs with saline, and then thoroughly dried for experiment. In order to minimize the effect of motion artifacts, the palm was fixed gently with adhesive tape on a fine-tuning translation stage. We moved the fiber rather than human hand to obtain spectrum data from the same area. After the raw spectrum data were obtained (as control), SA and SA@azone mixing solutions with different concentrations were topically applied onto the skin surface of palm and then spectra were acquired at the time intervals of 0, 20, 40, 60 and 80 min, respectively. The probe tip of optical fiber was kept close (~0.1 mm) to the tissue surface to avoid ambient light entrance of the detection system and reduce the light emission loss.

For individual experiment, the measurement repeated three times, and the average value was obtained with integration time of 4000 ms for error calibration. The program measurement takes ~10 ms to obtain optimal data, which was enough to prevent saturation and disregard noise. The relative humidity was maintained at 55 ± 10%. During the spectroscopic measurement, the changes of reflectance induced by SA and SA@azone mixing solutions at different concentrations were quantitatively evaluated by calculating the decrease in diffuse reflectance at 20, 40, 60 and 80 min, respectively. The reduction of diffuse reflectance Δ*R* was calculated through the degree of change to obtain spectrum of skin tissue at different time intervals 20, 40, 60 and 80 min after treatment with SA and SA@azone by following equation^36^.


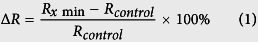


where *R*_*control*_ and *R*_*x*min_ were the measured diffuse reflectance at the wavelengths of 420, 540 and 580 nm, 20, 40, 60, and 80 min after treatment SA and SA@azone mixing solutions at different concentrations, respectively. The reason for choosing 420, 540 and 580 nm for the assessment of reflectance is because the SA and SA@azone causes the greatest changes of optical properties of the tissue at these three wavelengths seen from Figs 1 and 2. Therefore, the wavelengths of 420, 540 and 580 nm were chosen to calculate reflectance decrease for assessing the CPE of SA and SA@azone.

### OCT Measurements

In this work, all the imaging measurements were carried out using a portable time-domain OCT system with a low-coherence broadband super luminescent diode (SLD) light source at the central wavelength of 1310 nm with a spectral bandwidth of 50 nm. The axial and transverse resolutions were 15 μm and 25 μm, respectively. The image depth in skin was 1–3 mm. The signaltonoise ratio(SNR) of this system was 100 dB. The details of this OCT system were described previously^37^. The OCT imaging procedures was similar to the spectroscopic measurement described above. To obtain a baseline value, the selected region of each palm was monitored for 8 to 10 min prior to treatment with SA and SA@azone mixing solutions at different concentrations. The imaging area by OCT was the same as that of the spectra measurements. After obtaining the OCT images of native palm skin (as control), SA and SA@azone mixing solutions at different concentrations were topically applied onto the palm. Then 2-D OCT images were acquired with five averaging of A-scans at the time intervals of 20, 40, 60, and 80 min, respectively.

Three repeated experiments for every SA and SA@azone group with three volunteers were performed. A curve displaying the distribution of light in depth (Fig. S4b) was created by averaging the A-scans in dotted box in Fig. S4a (~1 mm) for speckle noise suppression. The data acquisition was acquired from Labview 7.2-D platform. OCT images were obtained in each experiment and stored in computer for further processing.

### Histology study

To confirm the structure difference of the skin after being treated with S_1_-S_4_ and S_1_@A-S_4_@A, hematoxylin and eosin (H&E) histology (10-fold magnification) of fresh porcine skin was implemented at a representative time point of 40 min (Fig. S5). The boundary of skin SC is very obvious as the red arrow indicated. Compared with Fig. S5b–i, Fig. S5a also shows a much denser and regular epidermis structure of skin. The epidermis texture/structure of the skin was destroyed after being treated with S_1_-S_4_ in Fig. S5(b–e). This phenomenon is attributable to the SA, which possesses the effect of keratolytic and desmolytic on skin. Furthermore, the structures of skin in Fig. S5(f–i) are much looser than those in Fig. S5(b–e) at the same time intervals. The changes on the structure and intercellular space of skin can be obviously observed from Fig. S5(j–l) as the red arrow indicated.

### Statistical Analysis

All the data were presented as mean ± standard error for results obtained from three independent trials unless otherwise indicated. A paired t-test statistical analysis was used to determine significance of differences in reflectance decrease and light penetration depth increase between SA alone and SA@azone treated groups. The data used for the analysis was confirmed to normally distribute by one sample Kolmogorov-Smirnov test, which were done by SPSS 16.0 software. And p < 0.05 was considered as statistically significant.

## Additional Information

**How to cite this article**: Zhao, Q. *et al*. Synergistic efficacy of salicylic acid with a penetration enhancer on human skin monitored by OCT and diffuse reflectance spectroscopy. *Sci. Rep.* **6**, 34954; doi: 10.1038/srep34954 (2016).

## Supplementary Material

Supplementary Information
