# Supplementary material for: Synergistic efficacy of salicylic acid with a penetration enhancer on human skin monitored by OCT and diffuse reflectance spectroscopy
Source: Sci Rep. 2016 Oct 10;6:34954. doi: 10.1038/srep34954 (PMC5056361; doi:10.1038/srep34954)
Supplement: Supplementary Information [file srep34954-s1.pdf]

Supporting Information

Synergistic efficacy of salicylic acid with a penetration enhancer on human skin monitored by OCT and diffuse reflectance spectroscopy

Qingliang Zhao[1], Cuixia Dai[2], Shanhui Fan[3], Jing lv[1], Liming Nie[1*]

[1]State Key Laboratory of Molecular Vaccinology and Molecular Diagnostics & Center for Molecular Imaging and Translational Medicine, School of Public Health, Xiamen University, Xiamen 361102, China

[2]School of science, Shanghai Institute of Technology, Shanghai 201418, China

[3]College of Life Information Science and Instrument Engineering, Hangzhou Dianzi University, Hangzhou 310018, China

[*]Corresponding author. Emails address: nielm@xmu.edu.cn (Liming Nie)

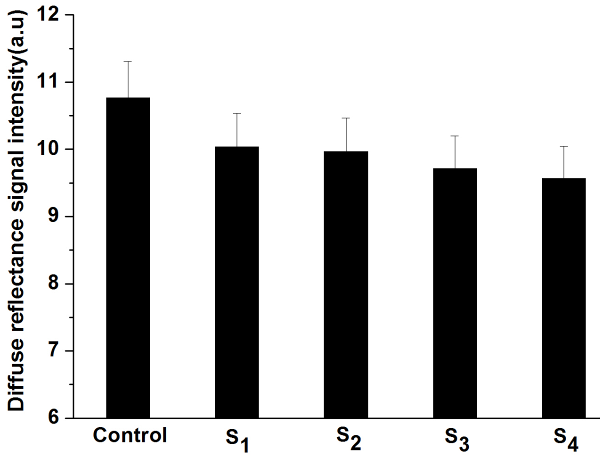

Figure S1. The DR signal intensity. The DR signal intensity change of the control and four groups (S_1-S_4) after 80 min treatment at 580 nm.

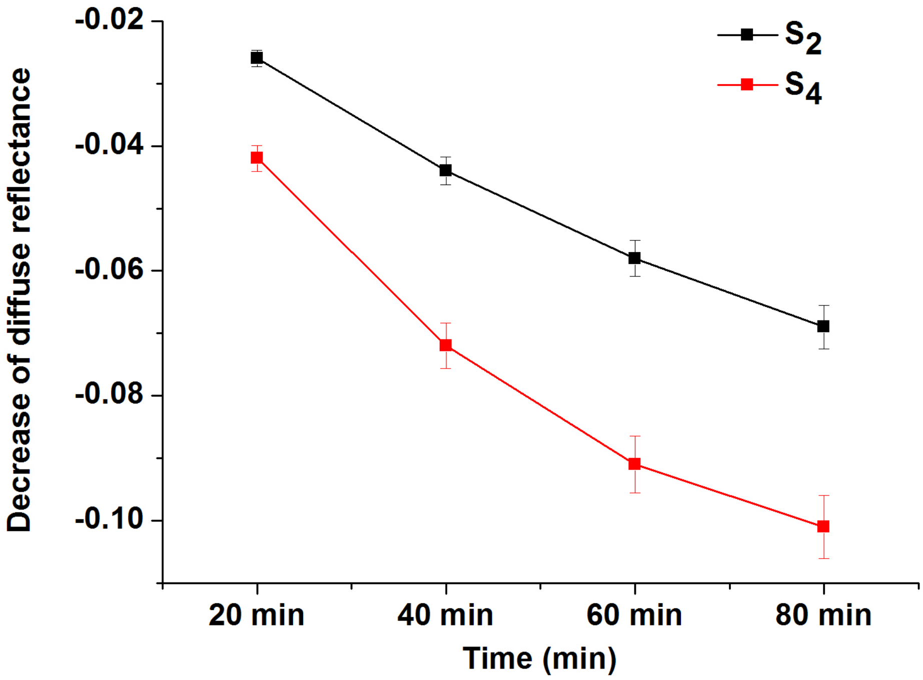

Figure S2. The decrease of DR intensity. The DR values decrease at different SA concentrations for S_2 and S_4 groups at 540 nm, respectively.

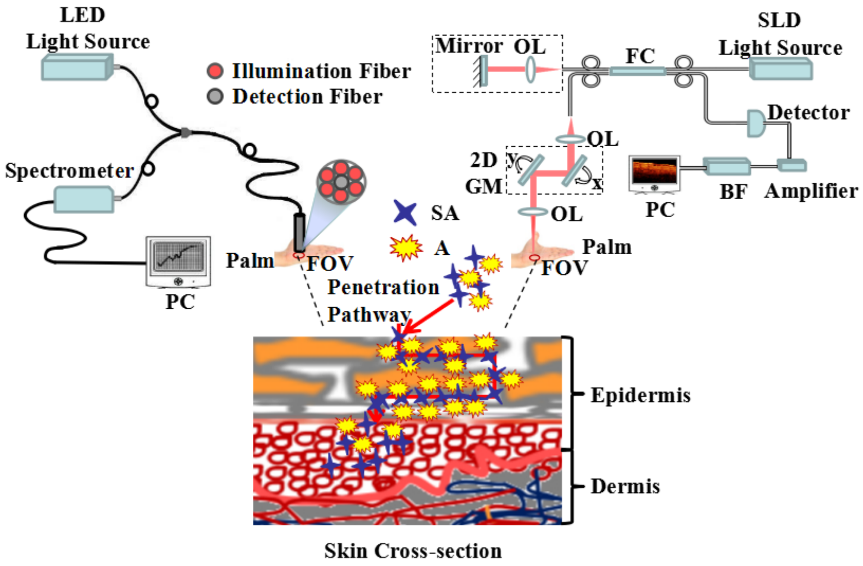

Figure S3. Schematic diagram of the DRS and OCT system. SLD: Super-luminescent Diodes, FC: Fiber Coupler, GM: Galvo Mirrors, OL: Objective Lens, PC: Personal Computer, BF: Bandpass Filter, FOV: Field of View, SA: Salicylic Acid, A: Azone.

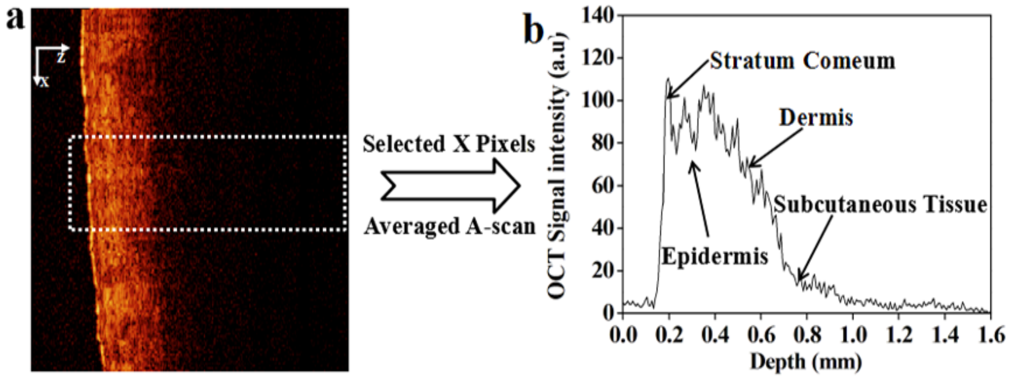

Figure S4. Extracted one-dimensional OCT signals. (a) A representative two-dimensional OCT image obtained from normal human skin. (b) The corresponding one-dimensional OCT signals.

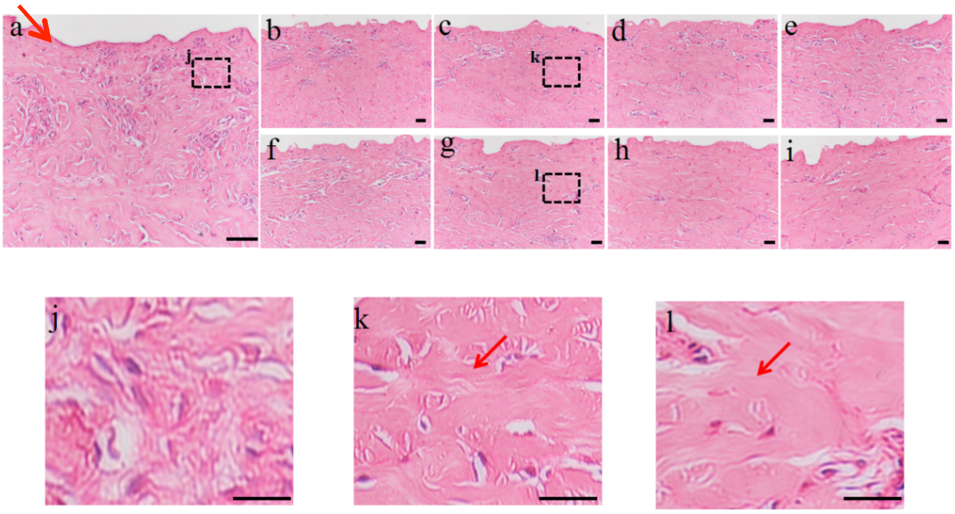

Figure S5. H&E histology images of porcine skin. (**a**) The histology image of porcine skin without treatment. (**b**)-(**e**) treated with S_1-S_4; (**f**)-(**i**) treated with S_1@A-S_4@A, respectively. (**j**), (**k**) and (**l**) magnifications of regions within the black dotted line boxes in (**a**), (**c**) and (**g**). Scale bar=200 μm in (**a**), 100 μm in (**b**)-(**i**), and 50 μm in (**j**), (**k**) and (**l**).
